# Supplementary material for: Rapid, label-free histopathological diagnosis of liver cancer based on Raman spectroscopy and deep learning
Source: Nat Commun. 2023 Jan 4;14:48. doi: 10.1038/s41467-022-35696-2 (PMC9813224; doi:10.1038/s41467-022-35696-2)
Supplement: Supplementary file 1 — Supplementary information [file 41467_2022_35696_MOESM1_ESM.docx]

**Supplementary information**

**Rapid, label-free histopathological diagnosis of liver cancer based on Raman spectroscopy and deep learning**

Liping Huang et al.

**Supplementary Fig. 1 Raman spectra of liver tissue obtained by lasers with different wavelengths.** Different laser wavelengths were compared, including 532 nm, 633 nm, and 785 nm. As a result, the shorter wavelengths 532 nm provided higher data quality and signal-to-noise ratio for Raman spectra. Source data are provided as a Source Data file.

**Supplementary Fig. 2 Typical Raman spectra collected from paracarcinoma (a) and carcinoma (b) tissues.** The variances among different Raman spectra indicate the heterogeneity of liver tumour tissue.

**Supplementary Fig. 3 Hierarchically-clustered heatmap of representative Raman intensities of liver tissue samples.** The heatmap was coloured based on Pearson's correlation coefficient (r), which lied between -1 and +1, -1 indicating total negative linear correlation, 0 indicating no linear correlation, and 1 indicating total positive linear correlation. Raman peaks with high correlation were pre-assigned to the same type of substance or chemical group. In most studies, the assignment of Raman peaks from complex biological samples was primarily based on previous literature. However, because of the differences in biological samples and the complexity of chemical functional groups, the Raman peak information might likely be assigned incorrectly, resulting in erroneous metabolic analysis results. In comparison, this study utilized hierarchical clustering analysis combined with Pearson’s correlation coefficient analysis to pre-discriminate the closely related Raman peaks, which could effectively reduce the bias caused by human judgment.

**Supplementary Fig. 4 The spectra difference among different liver cancer cell differentiation groups.** (a) The spectral difference between well and moderately differentiated groups. (b**)** The spectral difference between moderately and poorly differentiated groups. Source data are provided as a Source Data file.

**Supplementary Fig. 5 Scatter distribution of serum alpha-fetoprotein (AFP) concentration in HCC patients.** HCC patients n=92. Centerline, median; upper and lower quartiles was indicated respectively. Serological test of AFP is the common method for the screening of HCC. However, under the AFP threshold of 200 ng/mL (yellow line), 25 of the 92 HCC patients in this study were positive, and the detection sensitivity was only 27.2%. Source data are provided as a Source Data file.

**Supplementary Fig. 6** **The ROC curve of the classification of the grade of microvascular invasion (MVI) based on Raman spectra and the VGG-16 model.** In all 120 patients with liver cancer, the MVI status of 84 patients was obtained, of which 61 were M0 (without MVI), and the remaining 23 were M1 or M2. The accuracy to differentiate M0 and M1/M2 is 66.9% based on the deep learning model and the ROC value is 0.694. Source data are provided as a Source Data file.

**Supplementary Fig. 7** Relative contents of representative phosphatidylcholines (a-b), nucleosides, bases (c), and saccharides (d) with significant differences between HCC tissues and adjacent tissues. The signal for each metabolite from adjacent tissue was set to 1. Data are presented as mean values ± SD. HCC tissues, n=25, adjacent tissues, n=25. Source data are provided as a Source Data file.


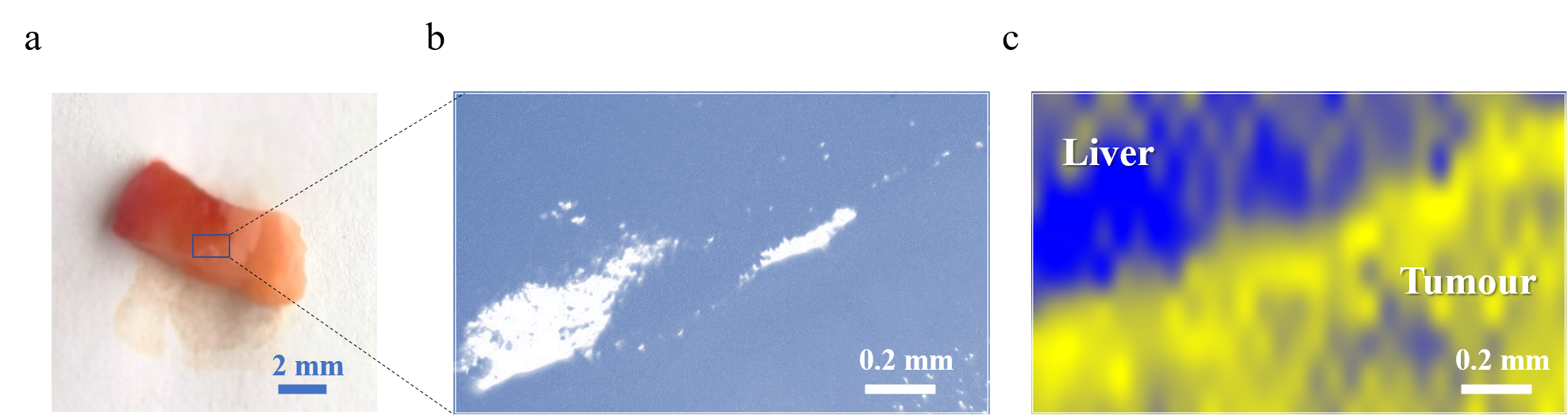


**Supplementary Fig. 8** A liver cancer tissue block (a), and its bright-field image (b) and corresponding Raman image (c) revealing the cancer boundary. Raman spectra were acquired with a 5× objective (NA = 0.12, WD = 14 mm), equipped with a 532 nm laser, with 2.5 mW cm^-2^ laser power and 2 s exposure time for each data point. The Raman scans were collected with a resolution of 50 μm and 100 μm in the x- and y-directions (horizontal axis and vertical axis), respectively.

**Supplementary Fig. 9 Raman imaging of various liver tissue slices. a–e,** The White light images of the mapping test area and SMCR reconstructed concentration maps of protein and lipids and their overlay image of normal liver tissue (a) and other tissue regions with typical morphologies, including tissues with cancerization (b), steatohepatitis (c), fibrosis (d), and connective tissue (e). To merge the two images, the minimum LUT value of yellow (lipids) was adjusted. All the scale bars are 10 µm.

**Supplementary Fig. 10 SMCR derived Raman image and corresponding 3D reconstruction images of various liver tissue slices. a–e,** SMCR derived Raman image and corresponding 3D reconstruction image from six z-stack (5 μm.) images of normal liver tissue (a) and other tissue regions with typical morphologies, including tissues with cancerization (b), steatohepatitis (c), fibrosis (d), and connective tissue (e). All the scale bars are 10 µm.

**Supplementary Fig. 11 Raman spectra of liver cancer collected in different depth. a** Raman spectra were acquired in different depth of liver tissue block, with a depth interval of 25 μm for each acquisition from the surface (0 μm) to 300 μm depth. **b** Depth-resolved Raman intensity contour plot of liver tissue. Source data are provided as a Source Data file.

**Supplementary Fig. 12 The Raman spectra of an isolated liver carcinoma tissue block obtained by portable Raman with/without sterile protective cover.** The protective cover had a weak effect on the measured Raman spectrum, with a slight reduction in the intensity of the Raman peak. Source data are provided as a Source Data file.

**Supplementary Fig. 13 The average Raman spectrum of liver cancer obtained by the confocal micro-Raman spectrometer and portable Raman system.** There were variances in the Raman peak positions measured by the two pieces of equipment, which might be largely attributed to differences between the two spectral devices. Source data are provided as a Source Data file.

**Supplementary Table 1. Clinical characteristics of the tested patients.**

|  | **Hepatocellular Carcinoma (HCC) (n=98)** | **Intrahepatic Cholangiocarcinoma (ICC) (n=22)** |
| --- | --- | --- |
| Age (yrs) | 58.1±12.2 | 60.8±13.1 |
| Male  Females | 84 (85.7%)  14 (14.3%) | 13 (59.1%)  9 (40.9%) |
| Alcohol consumption ^a^  no drinking  < 28 g/day  ≥28 g/day | 59 (60.2%)  19 (19.4%)  20 (20.4%) | 16 (72.7%)  2 (9.1%)  4 (18.2%) |
| Hepatitis B Virus (HBV)  Hepatitis C Virus (HBV) | 64 (65.3%)  0 | 5 (22.7%)  0 |
| Hepatic Cirrhosis | 72 (73.5%) | 5 (22.7%) |
| Steatohepatitis | 20 (20.4%) | 6 (27.3%) |
| Stage | Early: 69  Advantage: 29 |  |
| Differentiation | Well: 27  Moderately: 40  Poorly: 31 |  |
| Alpha-Fetoprotein (AFP) (ng/mL) | Ave: 2939.2±10231.7  Median: 16.3 | Ave: 56.9±179.0  Median:3.20 |

^a^ refers to the *“Dietary Guidelines for Americans 2020-2025”*

**Supplementary Table 2. The peak position and assignments of the representative Raman vibrational modes of the liver cancer tissue and adjacent non-tumour tissue samples.**

| **Position** | **Vibrational mode** | **Major assignment** |
| --- | --- | --- |
| 674 | Ring breathing | DNA base guanine |
| 749 | symmetric ring breathing | Tryptophan |
| 835 |  | Saccharide |
| 974 | Ribose vibration & C-C backbone | Nucleic acids and proteins |
| 1003 | CH_3_ deformation or ring breathing | Carotenoids and phenylalanine |
| 1081 | Phosphate vibrations | Phospholipids and nucleic acids |
| 1130 | C-C stretching | Fatty acids |
| 1156 | C-C, C-N stretching | Carotenoids and proteins |
| 1212 | C-C_6_H_5_ stretching | Tyrosine and phenylalanine |
| 1304 | CH_2_ twisting, wagging | Collagen and phospholipids |
| 1336 | CH_3_CH_2_ twisting | Nucleic acids and collagen |
| 1356 | C=C stretching | Tryptophan, adenine, and guanine |
| 1393 | CH rocking | Tryptophan |
| 1446 | CH_2_ bending | Lipids and proteins |
| 1519 | C-C, C=N stretch | Carotenoids and proteins |
| 1547 | C=C stretching | Tryptophan |
| 1586 | C=C bending | Phenylalanine |
| 1602 | C=C in-plane bending | Tyrosine |
| 1637 | C=O stretching | Amide I band (α-helix and β-structure) |

**Supplementary Table 3. Comparison of the capability of the VGG model and machine learning algorithms to identify different pathological types of liver tissue.**

|  | Model | Accuracy (%) | Sensitivity (%) | Specificity (%) |  |
| --- | --- | --- | --- | --- | --- |
|  |  |  |  |  |  |
| Carcinoma | PLS-DA | 84.8 | 95.2 | 73.3 |  |
|  | XGboost | 87.9 | 91.8 | 83.5 |  |
|  | Random forest | 86.4 | 87.5 | 85.2 |  |
|  | **VGG-16** | **92.6** | **90.8** | **94.6** |  |
|  |  |  |  |  |  |
| HCC | PLS-DA | 76.9 | 93.5 | 10.5 |  |
|  | XGboost | 77.8 | 86.8 | 42.0 |  |
|  | Random forest | 77.0 | 79.0 | 69.0 |  |
|  | **VGG-16** | **82.4** | **82.6** | **81.5** |  |
|  |  |  |  |  |  |
| Advanced stage | PLS-DA | 63.3 | 4.2 | 94.6 |  |
|  | XGboost | 73.8 | 31.5 | 93.0 |  |
|  | Random forest | 75.5 | 75.4 | 75.5 |  |
|  | **VGG-16** | **78.3** | **65.8** | **84.1** |  |
|  |  |  |  |  |  |
| Poor | PLS-DA | 76.5 | 52.4 | 87.5 |  |
|  | XGboost | 69.6 | 24.0 | 90.4 |  |
|  | Random forest | 71.1 | 71.6 | 70.9 |  |
|  | **VGG-16** | **72.3** | **70.8** | **72.9** |  |

**Supplementary Table 4. Comparison of diagnostic results of liver cancer based on traditional imaging modalities and Raman spectroscopy in this study.**

| Pathological categories | Diagnostic results | References |
| --- | --- | --- |
| HCC/ICC | misdiagnosis rate of HCC for ICC patients with no risk factors for HCC was 24.3% (36 ICC) by CT and MRI | *World J. Surg.* 2020, 44, 3862-3867 |
|  | misdiagnosis rate of HCC for ICC patients with any risk factors for HCC was between 20.0 and 33.3% (54 ICC) by CT and MRI |  |
| HCC/ICC | misdiagnosis rate of HCC was 52% (25 ICC) by CEUS, 4.2% (24 ICC) and 9.1% (11 ICC) by CT and MRI | *Liver Int.* 2013, **33**, 771-779 |
| High-grade and low-grade HCC | accuracies are 60-70% (170 patients) based on non-contrast-enhanced MRI | *Eur. Radiol.* 2019, 29, 2802-2811 |
|  | an accuracy of 53.33% (297 subjects) based on contrast-enhanced CT | Eur. Radiol. 2020, 30, 6924-6932 |
| HCC | the sensitivities of US were 63-65% | Aliment. Pharmacol. Ther. 2009, 30, 37-47 |
| HCC | the sensitivities of dynamic CT and MRI were 63%-76% and 77%-90%, and the specificities were 87%-98% and 84%-97% | *World J. Gastroenterol.* 2018, 24, 2348-2362 |
| Per-lesion for nodular HCC | sensitivity of MRI for nodular HCC of all sizes is 77%–100%, while that of CT is 68%–91%  per-lesion sensitivities are 100% for both modalities for nodular HCCs larger than 2 cm, 44%–47% (MRI) and 40%– 44% (CT) for 1–2 cm HCCs  and 29%–43% (MRI) and 10%–33% (CT) for HCCs smaller than 1 cm | *Radiology* 2014, 273, 30-50, *Hepatology* 2003, 38, 1034-1042, and *Gut* 2010, 59, 638-644 |
| Liver cancer / paracancer tissues | sensitivity 90.8%  specificity 94.6%  accuracy 92.6% | in this study |
| HCC / ICC tissues | sensitivity 82.6%  specificity 81.5%  accuracy 82.4% |  |
| Early / Advantaged stage | sensitivity 84.1%  specificity 65.5%  accuracy 78.3% |  |
| Well and moderate / poor grade | sensitivity 72.9%  specificity 70.8%  accuracy 72.3% |  |

**References**

1. Cheng, N. et al. Pre-operative Imaging Characteristics in Histology-Proven Resected Intrahepatic Cholangiocarcinoma. *World J. Surg.* **44**, 3862-3867 (2020).

2. Galassi, M. et al. Patterns of appearance and risk of misdiagnosis of intrahepatic cholangiocarcinoma in cirrhosis at contrast enhanced ultrasound. *Liver Int*. **33**, 771-779 (2013).

3. Wu, M. et al. Predicting the grade of hepatocellular carcinoma based on non-contrast-enhanced MRI radiomics signature. *Eur. Radiol*. **29**, 2802-2811 (2019).

4. Mao, B. et al. Preoperative prediction for pathological grade of hepatocellular carcinoma via machine learning-based radiomics. *Eur. Radiol*. **30**, 6924-6932 (2020).

5. Singal, A. et al. Meta-analysis: surveillance with ultrasound for early-stage hepatocellular carcinoma in patients with cirrhosis. *Aliment. Pharmacol. Ther*. **30**, 37-47 (2009).

6. Jiang, H. Y. et al. Noninvasive imaging of hepatocellular carcinoma: From diagnosis to prognosis. *World J. Gastroenterol*. **24**, 2348-2362 (2018).

7. Choi, J. Y., Lee, J. M. & Sirlin, C. B. CT and MR imaging diagnosis and staging of hepatocellular carcinoma: part II. Extracellular agents, hepatobiliary agents, and ancillary imaging features. *Radiology* **273**, 30-50 (2014).

8. Burrel, M. et al. MRI angiogical CT for detection of HCC prior to liver transplantation: an explant correlation. *Hepatology* **38**, 1034-1042 (2003).

9. Sangiovanni, A. et al. The diagnostic and economic impact of contrast imaging techniques in the diagnosis of small hepatocellular carcinoma in cirrhosis. *Gut* **59**, 638-644 (2010).
