# Supplementary material for: Rapid, label-free histopathological diagnosis of liver cancer based on Raman spectroscopy and deep learning
Source: Nat Commun. 2023 Jan 4;14:48. doi: 10.1038/s41467-022-35696-2 (PMC9813224; doi:10.1038/s41467-022-35696-2)
Supplement: Supplementary file 3 — Description of Additional Supplementary Files [file 41467_2022_35696_MOESM3_ESM.docx]

**Description of Additional Supplementary Files**

**Supplementary Data 1.** Non-targeted metabolomics results based on liquid chromatography-mass spectrometry (LC-MS) in both positive and negative electrospray ionization source (ESI^+^ and ESI^-^) modes.

**Supplementary Data 2**. Identification of 108 significantly differential metabolites between HCC tissues and adjacent non-tumour tissues by liquid chromatography-mass spectrometry (LC-MS).
